# DOES ONSET OF CHRONIC CONDITIONS MODERATE THE IMPACT OF RELATIONAL LIFE EVENTS ON DEPRESSED MOOD?

**DOI:** 10.1093/geroni/igz038.970

**Published:** 2019-11-08

**Authors:** Aaron M Ogletree, Rosemary Blieszner, Rachel Pruchno, Jyoti Savla, Laura P Sands

**Affiliations:** 1 American Institutes for Research, Washington, District of Columbia, United States; 2 Virginia Tech, Blacksburg, Virginia, United States; 3 Rowan University, Stratford, New Jersey, United States

## Abstract

More than 62% of adults aged 65+ have more than one chronic condition; this number increases to more than 82% for those 85+. Older adults simultaneously experience changes in their relationships due to negative relational life events, including illness, injury, or death of a loved one. Stressors occurring in tandem can overload psychological resources and increase risk for poor mental health. Informed by the stress process model, we assessed the influence of relational life events on depressive symptoms over time and evaluated the moderating effects of chronic condition onset. Self-reports of four stressful life events, five chronic conditions, and depressive symptoms as measured by the CES-D came from 2,948 older adults participating in the ORANJ BOWL panel. Using longitudinal multilevel mixed effect modeling, we examined trajectories of depressive symptoms across three waves. While depressive symptoms increased over time, they were greater for people who experienced more relational life events and the onset of more chronic conditions. Participants who reported experiencing all four relational life events but no chronic conditions had an average CES-D score of 5.28 (p<.0001); average CES-D score increased to 12.72 (p<.0001) for those who reported four life events and the onset of four or more new chronic conditions during the study period. In summary, chronic condition onset moderated the relationship between life events and depressive symptoms. Findings highlight the need for practitioner awareness of increased mental health risks for people experiencing stressors in multiple domains of life.

